# Suppression of Glucocorticoid Response in Stressed Mice Using 50 Hz Electric Field According to Immobilization Degree and Posture

**DOI:** 10.3390/biology11091336

**Published:** 2022-09-09

**Authors:** Shinji Harakawa, Takuya Hori, Takao Hiramoto, Takaki Nedachi, Toshikazu Shinba, Hiroshi Suzuki

**Affiliations:** 1 Bio-Self-Regulating Science Laboratory, Obihiro University of Agriculture and Veterinary Medicine, Obihiro 0808555, Japan; 2 Hakuju Institute for Health Science, Tokyo 1510063, Japan; 3 Department of Psychiatry, Shizuoka Saiseikai General Hospital, Shizuoka 4228527, Japan

**Keywords:** stress, endocrine response, extremely low frequency, electrical stimulation

## Abstract

**Simple Summary:**

With an increasing demand for electricity and electrical equipment, humans are routinely and unintentionally exposed to electric fields (EFs); no significant adverse effects have been identified from exposure to EFs, but slight physiological effects are known to occur. There are, however, methods and devices that expose subjects to EFs for medical purposes. The effects of such methods are not strong and primarily involve the physical properties of EFs, which are invisible and easily disturbed, yet the mechanisms of the biological effects of such EFs have not been identified. We have developed a simple experimental system that allows us to reproducibly observe the inhibitory properties of EFs on the glucocorticoid response to added stress. Using this system, we have revealed the biological effects of EFs. This study has shown that human body posture may affect action of the EF and that it is important to adjust the degree of immobilization in order to capture the effect of the EF. The results of this study provide useful information not only for medical applications of EFs but also for the assessment of risks to health.

**Abstract:**

Various studies on immobilized BALB/c mice to evaluate changes in hormone levels associated with stress responses have advanced the characterization of multiple aspects of the biological actions of extremely low-frequency (ELF) electric fields (EFs). In this study, we aimed to investigate the effect of mouse posture on its stress responses and evaluate the importance of adjusting the stress degree in the model. Mice were immobilized inside centrifuge tubes and exposed to an ELF EF generated between parallel plate electrodes. Blood was collected under anesthesia immediately after EF exposure, and plasma glucocorticoids were assayed. The inhibitory effects of EFs on glucocorticoid elevation by immobilization were reproduced regardless whether mice were in the abdominal or lateral recumbent position, for the EF vector delivered to mice through the sagittal or frontal plane. The effect of ELF EF was reproduced in moderately and mildly stressed mice but not in severely immobilized mice. Hence, adjusting the stress degree is critical to the reproducibility of the results for this model. We characterized the effects of ELF EF on homeostasis, including the stress response, and provided valuable information for the scientific evaluation of the biological risks and medical applications of ELF EF.

## 1. Introduction

With the widespread use of electricity in domestic and industrial settings, it has now become necessary to investigate the biological effects of exposure to extremely low-frequency (ELF) electric fields (EFs), especially those fields generated at power-line frequencies [1,2,3,4]. In addition, ELF EFs are being used for non-contact and non-pharmacological medical applications in countries such as Japan, China, Korea, and Taiwan [2,5,6,7,8,9,10,11]. Several well-established therapeutic methods and devices using EF, including transcutaneous electrical nerve stimulation and vagal nerve stimulation, are used worldwide for treating depression, epilepsy, dementia, and pain. Electric currents induced in vivo and the perception of EF at the skin surface can induce cellular and humoral responses in certain organisms; however, the mechanisms of the biological effects induced by EFs are not well understood [1,2,3,12,13,14,15,16,17,18,19,20,21]. This is because the effects of EFs are not clear and involve the physical properties of these invisible and easily disturbed fields. Thus, understanding the biomedical effects of ELF EFs is necessary for human health. In addition, for ELF magnetic fields, the key interaction mechanism is the induction of EFs in the body, which may have the potential to produce diverse biological or health effects depending on their strength. On the other hand, for EFs at power-line frequencies, the coupling to the induced Efs in the body should be weaker, while the surface electric charge effects on the body may be more prominent [22].

To unveil biological effects of EF exposure, a reproducible evaluation system is needed. We have previously reported biological effects of ELF EF exposure for alleviating pain in the musculoskeletal system or of undefined origin as well as insomnia [7,8]. The bone density of humans exposed to EF therapy is higher than those of age-matched humans, and it increases with the treatment duration, as reported in [23]. The possibility of using electroencephalography and heartrate variability in humans to evaluate EF-induced changes has become clear [10]. Moreover, ELF EF can modulate energy metabolism [24] and intracellular calcium ion kinetics [25,26] as well as the endocrine [27,28] and immune systems [29]. However, EFs are easily influenced by external disturbances, and their biological evaluation requires a simple, robust, and fast system.

We developed an evaluation system that uses the glucocorticoid (GC) response of mice to EFs, generated using parallel plate electrodes. Using this system, we found the suppressive effect of EF exposure on the elevation of stress hormones during restraint, and the effect reproducibility was almost 100% in follow-up studies [27]. Corticosterone, the main GC produced by the adrenal gland, regulates the expression of the corticotropin-releasing factor gene or proopiomelanocortin gene [30]. An increase in the GC level can accompany an acute stress response as an adaptive mechanism [31,32]. In our system, immobilization and EF treatment were completed within 30–60 min, suggesting high reproducibility to assess unclear biological effects of EFs. The effects of EFs depend on the intensity (in kilovolts per meter) and exposure duration [33] as well as on the configuration of the EF exposure system [34]. For example, the immobilization-induced GC levels are significantly low in mice exposed to an EF of 1 kV/100 mm for 60 min but not in mice exposed to 0.5 kV/50 mm or 2 kV/200 mm, despite these systems generating a similar EF strength [34]. Additionally, the suppressive effect of EF exposure depends on the body surface area or portion exposed to the EF [28] and is independent of sex and age [35]. The evaluation system used in this study can elucidate the exact biological effects of power-line-frequency EF qualitatively and quantitatively.

As the relation between GC reduction and ELF EF becomes to be better understood, not only the biohazards but also the potential medical applicability of ELF EF can be studied based on the diverse functions of GCs [36,37,38,39,40,41]. For instance, the bone density of humans exposed to EF for therapeutic purposes is higher than that of humans of the same age without exposure, and the bone mineral density increases with a longer EF treatment [23]. Because osteoporosis is caused by the excessive production of GCs [42], the regulation of GC levels by ELF EF may be involved in its mechanism. Furthermore, ELF EFs seem effective in alleviating musculoskeletal and unexplained pain and insomnia [8]. Because GCs modulate inflammation [37,38,39,40,41], ELF EFs may influence stress and tissue damage involving inflammatory events [27]. Significantly understanding the relation between plasma GC concentrations and ELF EFs may lead to novel medical applications. The EF effects on stress responses [27,28,33] and heartrate variability [10] suggest that EFs can contribute to homeostasis. If the effects of ELF EFs on stress responses demonstrate homeostatic properties, they can be used as a shallow intervention to possibly support health, prevent illness, improve social productivity, and prevent or alleviate medical cost pressures without interfering with the work of practicing physicians.

Based on the high reproducibility of the effects induced by ELF EFs on stress responses, we have been characterizing the biological action of ELF EFs, potential for adverse effects on living organisms including humans, specification of EFs suitable for the medical efficacy, and influence of the external environment in the effects by using the developed system. In this study, we investigated the influence of subject posture on the suppressive effect of ELF EFs related to the stress response and the significance of the immobilization strength in the evaluation system using an immobilization model in BALB/c mice. The investigation of the involvement of a subject’s posture in the inhibitory action of electric fields in response to stress is not only related to the safety of electric fields but also to the specification of medical applications of the fields. This investigation may reveal information concerning the design of electric fields and may help advance our understanding of the coupling between electric fields and the body.

## 2. Materials and Methods

### 2.1. Animals

Mice, eight-week-old male BALB/c (Charles River Japan, Kanagawa, Japan), were maintained in a specific pathogen-free environment at 24 ± 1 °C and 50 ± 10% humidity with daily artificial illumination (12 h light/12 h dark cycle with lights on from 07:00–19:00). The animals had free access to standard laboratory chow (CE-2; CLEA, Tokyo, Japan) and water, except for the period during EF exposure and immobilization. The acclimatization period was two weeks.

All animal experiments and procedures were carried out in accordance with the Guiding Principles for the Care and Use of Research Animals of Obihiro University of Agriculture and Veterinary Medicine, Japan. The protocol was also approved by the Committee on the Ethics of Animal Experiments of Obihiro University of Agriculture and Veterinary Medicine (permit number 18–95, 19–162, 20–124).

### 2.2. EF-Treatment System

**Figure 1:**
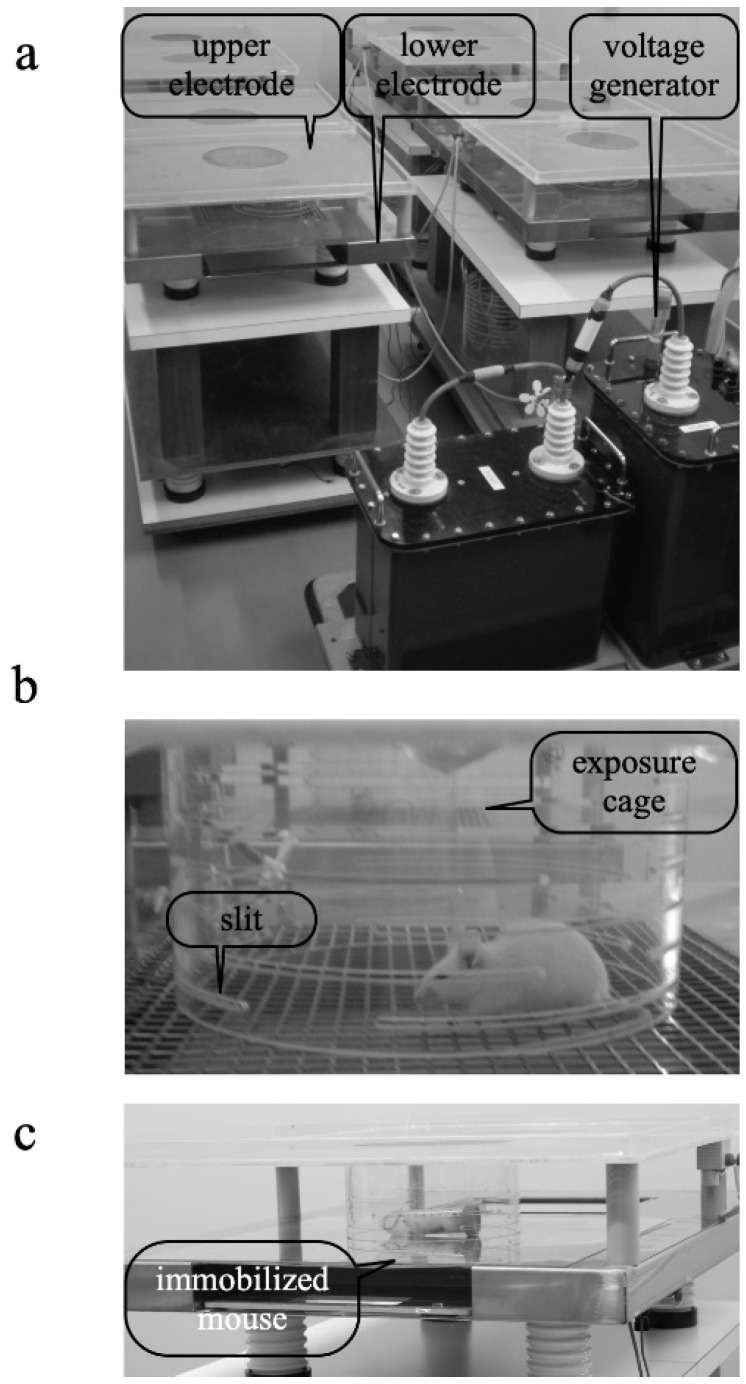
Appearance of EF exposure system. (**a**) High-voltage generator and parallel plate electrodes. (**b**) EF exposure cage with 100 mm × 5 mm slits spaced at 5 mm. (**c**) Centrifuge tube used to immobilize mice.

The EF-treatment system is described in detail in our previous papers [27,28,33]. Here it is briefly described. The system consisted of three main parts: a high-voltage transformer unit and a plastic cage held by two stainless-steel electrodes above and below (a, b). The cylindrical cage had slits at 5 mm intervals (Figure 1b) to prevent the effects of smudges (from feces or saliva) on the formation of a stable EF. During the experiment, the temperature in the cages was maintained at 24 ± 1 °C and humidity between 45% and 55%.

In the cage, mice in the EF-treated groups were exposed to an EF of 10 kV/m for 1 h generated by loading 1 kV at 50 Hz to the upper electrode and grounding the lower electrode. The EF intensity applied to the cage had an error of ±4% outside the exposure cage and ±0.1% inside the cage.

To assess the MF intensity in the field-treatment area of each mouse to the EF, a portable magnetic field meter (TMM-1; Electric Power Engineering Systems, Kanagawa, Japan) was used. The magnetic field intensity was approximately 0.012 ± 0.004 μT for a 50 Hz 10 kV/m EF generated in space. The information on magnetic field contamination is highly important to ensure its quality in studies aimed to investigate the biological effects of only electric fields, not including magnetic fields, and this value is considered to be sufficiently low.

### 2.3. Immobilization Stress

Each mouse was separately immobilized in a 50 mL centrifuge tube (Nippon Genetics, Tokyo, Japan) and placed on the lower electrode to induce stress (Figure 1c) [27,28]. The centrifuge tube for immobilization had a gap to allow breathing, and the lid had a hole with an approximate diameter of 3 mm. The GC increase was a stress response immediately after restraint and peaked approximately 30 min later. If immobilization continued after reaching the peak GC, preliminary studies indicated that the levels gradually decrease over time. To use the changes in GC level as an indicator of the response to stress, we reasoned that GC data should be obtained 30 min after the stress treatment onset.

### 2.4. Effect of Mouse Posture on EF-Induced Suppression of Stress Response

**Figure 2:**
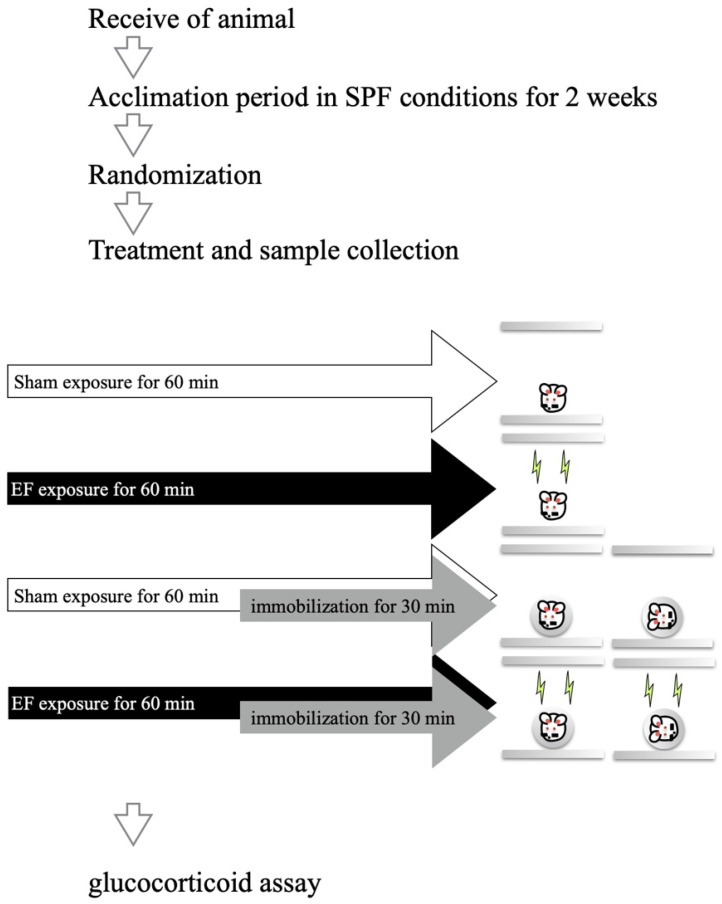
Experimental design to test the effects of different mice postures when immobilized and/or exposed to EF. The 96 mice were divided into six groups (*n* = 16 per group): control (stress(−)/EF(−)), EF-alone (stress(−)/EF(+), two immobilization-alone (stress(+)/EF(−)), and two cotreatment (stress(+)/EF(+)) groups. The mice were placed in abdominal or lateral recumbent position. In the abdominal and lateral recumbent positions, the EF vector was perpendicular to the frontal and sagittal planes of the mouse, respectively.

To examine the influence of mouse posture and EF on plasma stress hormones (i.e., GCs) in immobilized mice, the 96 mice were divided into (1) control (stress(−)/EF(−), *n* = 16), (2) EF-alone (stress(−)/EF(+), *n* = 16), (3) immobilization-alone (stress(+)/EF(−), *n* = 32), and (4) cotreatment (stress(+)/EF(+), *n* = 32) groups (Figure 2). The immobilization-alone and cotreatment groups were divided into two subgroups according to the posture during immobilization (*n* = 16 per group): abdominal or lateral recumbent position. In the abdominal position, the EF vector with respect to the mouse was perpendicular to the frontal plane. In the lateral recumbent position, the vector was perpendicular to the sagittal plane. Mice in the EF-treatment groups were exposed to the same EF (10 kV/m at 50 Hz for 60 min), and those in the cotreatment group were immobilized during the second half (30 min) of the EF exposure period [33]. Mice in the sham group were housed in the EF cage for 60 min without EF exposure (i.e., 0 V/m). The measurement of GC level is described in our previous papers [27,28,33].

### 2.5. Immobilization Degree and EF Suppression of Stress Response

**Figure 3:**
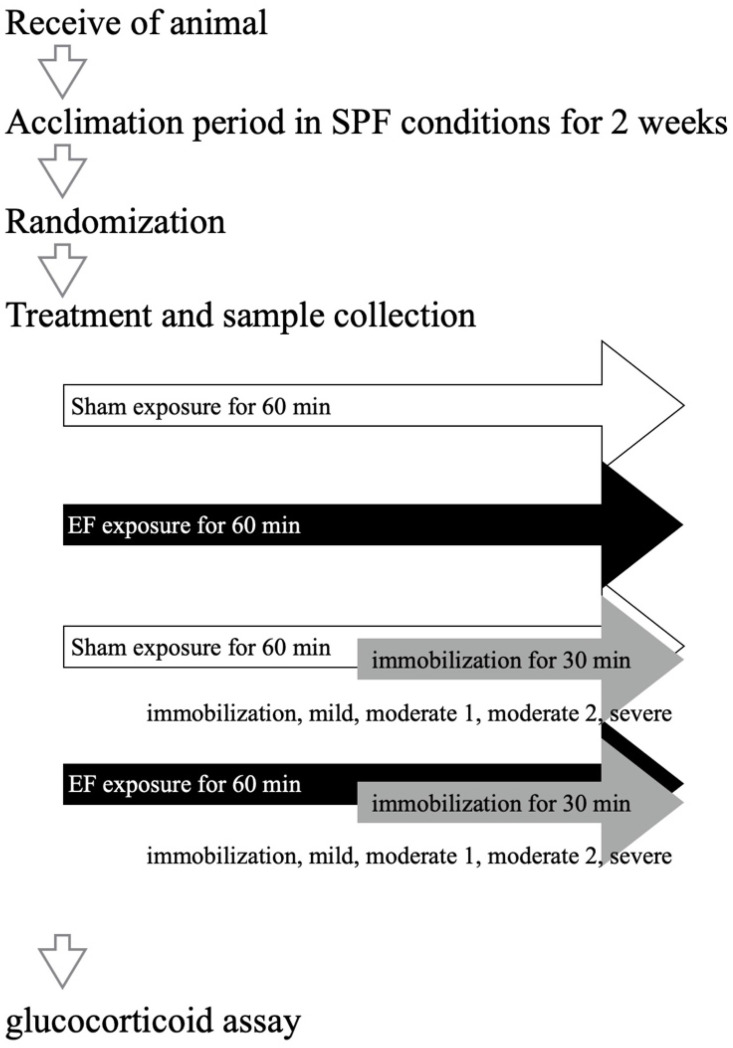
Experimental design to assess EF suppression of stress response according to immobilization degree. We divided 64 mice into eight groups: control (stress(−)/EF(−), *n* = 8), EF-alone (stress(−)/EF(+), *n* = 8), immobilization-alone (stress(+)/EF(−), *n* = 24), and cotreatment (stress(+)/EF(+), *n* = 24) groups. Three stress degrees were arranged according to volume for mouse immobilization (Figure 3). The volumes for moderate, mild, and severe stress degrees were 40, 48, and 32 mL, respectively.

To examine the influence by immobilization degree on the EF suppression of the stress response, we divided 60 mice into 10 groups: control (stress(−)/EF(−), *n* = 8), EF-alone (stress(−)/EF(+), *n* = 8), immobilization-alone (stress(+)/EF(−), *n* = 24), and cotreatment (stress(+)/EF(+), *n* = 24) groups. Three stress degrees were arranged according to the volume holding the mouse immobilized (Figure 3). The volume for moderate stress was 40 mL, as in our previous studies [27,28,33], whereas those for mild and severe stress were 48 and 32 mL, respectively. Those volumes were adjusted by inserts obtained from a screw container (Watson Bio Lab, Tokyo, Japan).

### 2.6. Statistical Analysis

The differences between groups were evaluated using two-way analysis of variance (ANOVA), and those between two groups were evaluated using Sidak’s multiple-comparisons test. The level of significance was set to *p* < 0.05. All statistical analyses were conducted using Prism Version 8 (GraphPad Software, La Jolla, CA, USA).

## 3. Results

### 3.1. Effect of Mouse Posture on EF-Induced Suppression of Stress Response

**Figure 4:**
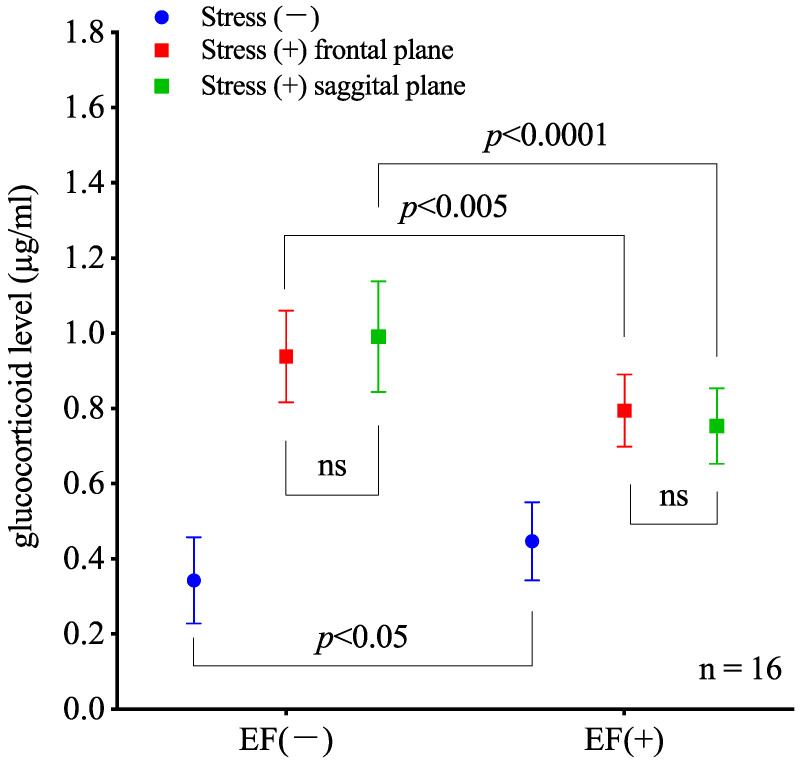
Evaluation of mouse postures when being immobilized and/or exposed to EF. For two-way ANOVA and main effect EF, F(DFn, DFd) = F(1, 90) = 15.47, *p* = 0.0002. For stress, F(1, 90) = 180.3, *p* < 0.0001. For interaction, F(1, 90) = 18.68, *p* < 0.0001. For Sidak’s multiple comparisons, the *p*-value between EF(−) and EF(+) is 0.0378 for stress(−), 0.002 for stress(+) in the abdominal position, and <0.0001 for stress (+) in the lateral recumbent position. Without stress, the GC level in EF-treated mice is significantly higher than that in mice not exposed to EF. The level is significantly lower in immobilized mice exposed to EF than in immobilized mice not exposed to EF regardless of the number of immobilizations. (ns, no significant difference).

The two-way ANOVA results indicate that immobilization significantly affects the GC levels (*p* < 0.0001; two-way ANOVA; Figure 4) and the EF treatment (or sham treatment) does not cause a significant change (*p* < 0.005). In addition, an interaction between immobilization and EF exposure is observed (*p* < 0.0001). Sidak’s multiple comparisons for post-hoc analysis show that the GC level in the stress(−)/EF(+) group is higher than that in the stress(−)/EF(−) group (*p* = 0.0378), and the GC level in the stress(+)/EF(+) group is lower than that in the stress(+)/EF(−) group (*p* = 0.002 in abdominal position and *p* < 0.0001 in lateral recumbent position, Figure 4). In the blood properties, no difference between the sham and EF-treated mice is observed.

### 3.2. Effect of Stress Degree on Suppressive Effect of EF

**Figure 5:**
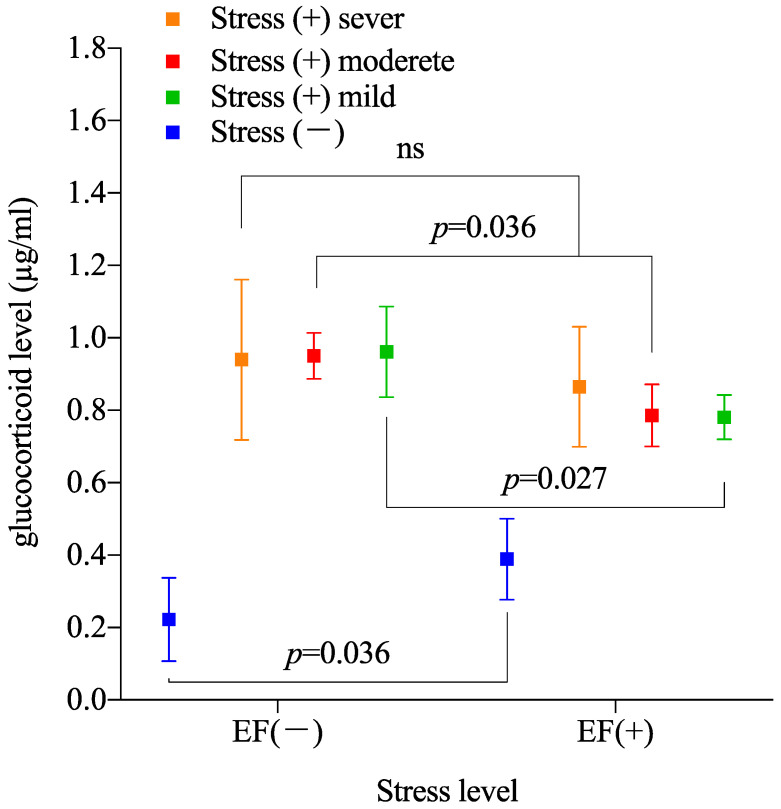
EF-induced stress suppression compared for different immobilization degrees. Two-way ANOVA was performed with presence or absence of EF and stress as main effect for six groups. For two-way ANOVA and main effect stress, F(2, 42) = 273, *p* = 0.7281. For EF, F(1, 42) = 13.17, *p* = 0.0008. For interaction, F(2, 42) = 0.7272, *p* = 0.4892. For Sidak’s multiple comparisons, the *p*-value between stress(−)/EF(−) and stress(−)/EF(+) is 0.0354 under moderate stress, 0.0295 under mild stress, and 0.2691 under severe stress. (ns, no significant difference).

The two-way ANOVA of the measured plasma GC level between stress(−)/EF(−), stress(−)/EF(+), moderate stress(+)/EF(−), and moderate stress(+)/EF(+) groups indicates significant differences for stress (*p* < 0.0001, Figure 5), whereas the EF treatment does not have an effect (*p* = 0.054), and their interaction is significant (*p* < 0.005). Sidak’s multiple comparisons demonstrate that the plasma GC level of the EF-treated groups is lower than that of the sham groups for moderate and mild stress degrees (moderate stress, *p* = 0.036; mild stress, *p* = 0.027; Figure 5). On the other hand, under severe stress, no significant difference is observed between the EF-treated and sham groups (*p* = 0.027).

## 4. Discussion

To evaluate the effects of EF exposure on mice immobilized with different postures, we induced stress by confinement in a centrifuge tube for 30 min. The plasma GC level in immobilized mice (i.e., stress(+)/EF(−) group) was approximately 2.7 times higher than that in the control (i.e., stress(−)/EF(−)) group, suggesting that immobilization activates the endocrine system of the pituitary–adrenocortical axis and/or sympathetic adrenomedullary system [31,43,44]. Plasma GC increases in male BALB/c mice subjected to both immobilization stress and exposure to a 50 Hz EF when voltage is delivered through the upper electrode of parallel plate electrodes, but the GC level was lower than that in the immobilization-alone group (Figure 4). These results are consistent with our previous observations [27,28,33].

The stress-reduction mouse model suggests an antistress effect of EF occurring in the abdominal and lateral recumbent positions (Figure 4). However, for the medical application of electric fields in society, we believe that posture should be carefully considered in further study. To verify, experiments with the electrodes rotating around the mouse in the abdominal position should be conducted.

Strong or prolonged stress is known to cause and contribute to many acute and chronic diseases [32,42]. GCs, the product of the hypothalamus–pituitary–adrenal axis, play a fundamental role in maintaining resting- and stress-related homeostasis, and influence physiological adaptive responses of an organism to stressors. Further research is needed to elucidate the mechanism by which ELF EF exposure suppresses the increase in GC levels; it is possible that ELF EFs act on the homeostasis regulating the stress response. In fact, we have observed effects on brain waves and heartrate variability of humans owing to EF exposure [10], and electromagnetic hypersensitivity associates electromagnetic fields with physical or mental problems [45,46]. Although the mechanism is not clear and negative views of causality exist, some people have certain health problems and believe that electromagnetic fields are their main cause. By limiting the scope to power-line-frequency EF, the effects of EF on homeostasis, including the autonomic or endocrine systems, may cause discomfort. Although no detailed studies have been conducted, this may be because some people who use EFs based on Japanese power supplies for therapeutic purposes show discomfort similar to that experienced through exposure to hot water or motion sickness, according to the author’s experience.

Considering the spillover effects on GCs, we can identify findings consistent with previous studies. Stress-induced adrenal GC increases, orthologous neuropeptides known as RFamide-related peptides that contribute to hypothalamic suppression of the reproductive function [36], and improvement in the copulation rate of C57BL/6J mice [47] may be related to EF-induced modulation of GCs. The bone density in men who routinely used EF therapy significantly exceeded the average in same-aged Japanese men, and the values increased with the duration of EF treatment [23]. GC-induced osteoporosis is a well-known metabolic response, and the EF-induced modulation of GCs may be related to it. The observations in [23,47] may indicate a cumulative effect of treatment based on ELF EFs. In addition, we have shown the effectiveness of ELF EF exposure in relieving musculoskeletal and unexplained pain and insomnia [8,23]. GCs reduce inflammation [37,38,39,40,41], and ELF EFs may act on the link between stress and tissue damage involving inflammatory events. As the relation between plasma GC levels and ELF EFs becomes clearer, the wide range of responses related to GCs may increase the medical applicability of such EFs.

We evaluated different immobilization strengths in the proposed system using a mice model. The effect of ELF EFs was reproducible in moderately and mildly stressed mice but not in severely immobilized mice (Figure 5). Thus, EFs likely become ineffective under excessive stress. Thus, although our model allows observing the biological effects of ELF EFs, adjusting the stress degree is critical for reproducibility. As the suppressive effect of ELF EFs on the immobilization-induced increase in GC can be reproduced, our model to assess biological responses elicited by EF exposure seems promising (Figure 4 and Figure 5). The proposed stress-loading model is one of few appropriate evaluation models for scientific understanding of the biological effects of ELF EFs. Unlike similar models, we can also adjust the immobilization degree to refine evaluations. In addition, based on the present results, the experimental system could be further improved by developing the study together with an index that shows a linear correlation with the stress intensity.

Our experimental model has some limitations. In the three experiments, an EF intensity of 10 kV/m was selected based on previous studies on EF intensities of 2.5–200 kV/m, in which 10 kV/m demonstrated the highest antistress effect of EF at 50 Hz [33]. In addition, EFs with intensities above 50 kV/m generate vibration and/or noise [33], whose effects are difficult to distinguish from those of the EF, undermining artifact removal at high EF intensities. This problem remains to be addressed and resolved to further study the effects of high-intensity EFs. Furthermore, placement of a 0.1 mm thick polypropylene sheet between the lower electrode and the animal as an insulator caused the suppression effect of EF on GC elevation due to restraint to disappear, but the effect reappeared as the thickness of the sheet increased [34]. Although the proposed system provides high reproducibility and can enable further investigation to understand the biological effects of ELF EF, additional developments are required for its application to humans.

Given the health risks and medical applications of ELF EFs, conflicting interpretations about the results and their magnitudes have appeared even in the same scope, such as the endocrine system considered in this study. In terms of health risks, both the International Commission on Non-Ionizing Radiation Protection and World Health Organization agree that additional experimental evaluations of ELF EFs are required [1,2,3,4]. Nevertheless, the inevitable biological variability, even if it is small, should be considered. For instance, electric shocks from a short circuit between a charged mouse and the exposure system have been reported to provoke a stress response in animals [48]; however, this phenomenon was not observed in our studies. Thus, it remains to be determined whether EFs alone can act as stressors, and unexpected effects of ELF EFs on evoked stress response should be unveiled.

## 5. Conclusions

We used a system that provides high reproducibility regarding plasma GC concentrations under the action of ELF EFs. Various experimental results suggested an action of ELF EFs on the homeostasis of mice. Stress suppression owing to EF exposure occurred regardless of the mouse posture or the EF vector and suggests flexibility in terms of posture or EF direction for risk assessment or medical applications. We also found that biological changes induced by EFs were difficult to identify under excessive stress. These results may deepen our understanding for risk assessment and preventive medical applications of ELF EFs toward their use in other species, including humans, and to treat GC-related diseases.

## Data Availability

We declare that all data supporting the findings of this study are available within the article.
